# Stable Extracellular RNA Fragments of *Mycobacterium tuberculosis* Induce Early Apoptosis in Human Monocytes via a Caspase-8 Dependent Mechanism

**DOI:** 10.1371/journal.pone.0029970

**Published:** 2012-01-09

**Authors:** Andrés Obregón-Henao, María A. Duque-Correa, Mauricio Rojas, Luis F. García, Patrick J. Brennan, Blanca L. Ortiz, John T. Belisle

**Affiliations:** 1 Grupo de Inmunología Celular e Inmunogenética, Universidad de Antioquia, Medellín, Colombia; 2 Mycobacteria Research Laboratories, Department of Microbiology, Immunology and Pathology, Colorado State University, Fort Collins, Colorado, United States of America; University of Delhi, India

## Abstract

The molecular basis of pathogen-induced host cell apoptosis is well characterized for a number of microorganisms. *Mycobacterium tuberculosis* is known to induce apoptosis and it was shown that live but not heat killed *M. tuberculosis* stimulates this biological pathway in monocytes. The dependence of this activity on live bacilli led us to hypothesize that products released or secreted by *M. tuberculosis* are the primary apoptotic factors for human monocytes. Thus, the culture filtrate of *in vitro* grown *M. tuberculosis* strain H37Rv was fractioned by conventional chromatography and the apoptosis-inducing activity of individual fractions was measured on human monocytes. The tests employed included measurement of cell membrane damage, caspase activation, and cytokine release. Small molecular weight RNAs of *M. tuberculosis* were recognized as the predominant apoptosis inducing factors. The RNA was comprised primarily of tRNA and rRNA fragments that stably accumulate in the culture filtrate during early log-phase growth. The RNA fragments signaled through a caspase-8 dependent, caspase-1 and TNF-α independent pathway that ultimately compromised the human monocytes' ability to control *M. tuberculosis* infection. These studies provide the first report of bacterial RNA inducing apoptosis. They also provide a foundation to pursue pathways for secretion or release of nucleic acids from *M. tuberculosis* and the impact of secreted RNA fragments on pathogenesis.

## Introduction

Apoptosis mainly occurs following activation of the caspase cascade by the mitochondrial or apical pathways [1], [2]. Mitochondrial release of organelle constituents such as cytochrome c and other macromolecules [3], activates the caspase cascade via pro-caspase-9. Alternatively the apical pathway is mediated by activation of pro-caspase-8 after cross linking of cell surface receptors belonging to the Tumor Necrosis Factor Receptor (TNFR) super family [4]. Both pathways converge to activate the executioner caspase-3 [1], [3]. Other alternative pathways designated as “type II” that do not rely on caspase 3 activity were described recently [5]. Cell death ensues after cleavage of the cell's cytoskeleton and intracellular enzymes involved in cellular homeostasis, leading to apoptosis hallmarks such as exposure of phosphatidylserine (PS) in the cell membrane, DNA fragmentation, and cell shrinkage [1], [2].

Apoptosis is a process associated with multiple infectious diseases including tuberculosis [6]. Several lines of evidence suggest that apoptosis is an important process occurring during mycobacterial infection [7]. Recently, caspase activation and TUNEL staining demonstrated that apoptosis is a major event occurring in the caseous necrosis of granulomas from *M. tuberculosis*-infected humans [8]. More importantly, *sst-1* and *slca11* (formerly *nramp-1*), the best characterized murine genetic loci determining susceptibility/resistance to mycobacterial infections, were associated with the degree of macrophage apoptosis [9]–[11]. It also is known that macrophage infection with live *M. tuberculosis* or stimulation with PPD activate both the apical and mitochondrial apoptotic pathways by inducing TNF-α production, caspase activation and calcium influx, [11]–[15]. In contrast dead *M. tuberculosis* and lipoarabinomannan (ManLAM) inhibit these pathways by inducing IL-10, activating Akt and blocking calcium influx [14]–[18].

Several mycobacterial molecules of diverse chemical nature were reported to induce apoptosis in various types of host cells. In particular, the 19 kilodalton (kDa) lipoprotein was shown to trigger murine and human macrophages apoptosis through TLR-2 via nitric oxide dependent and independent pathways [19]–[22]. Glycolipids such as trehalose dimycolate (TDM) and lipomannan (LM) from *M. tuberculosis* also activate apoptosis [23]–[25]. TDM induces apoptosis of murine natural killer and naïve T cells [23], [24], while LM targets human macrophages [25]. Contrary to the consistent finding that macrophage apoptosis requires infection with live, metabolically active mycobacteria [11], the aforementioned mycobacterial molecules are cell wall associated products purified from inactivated cells. However, a systematic purification of secreted products released by actively growing *M. tuberculosis* that promote apoptosis has not been performed.

To further address this issue we purified the major apoptosis-inducing product released by virulent *M. tuberculosis* strain H37Rv, and unexpectedly discovered this primary apoptotic factor was stable mycobacterial RNA fragments and not a protein or lipid. Additional experimentation revealed the nature of these stable RNA molecules and that their induction of apoptosis was via a caspase-8 dependent, and TNF-α and caspase-1 independent mechanism that affected the ability of human monocytes to control mycobacterial infection.

## Results

### Purification and identification of apoptosis inducer

**Figure 1:**
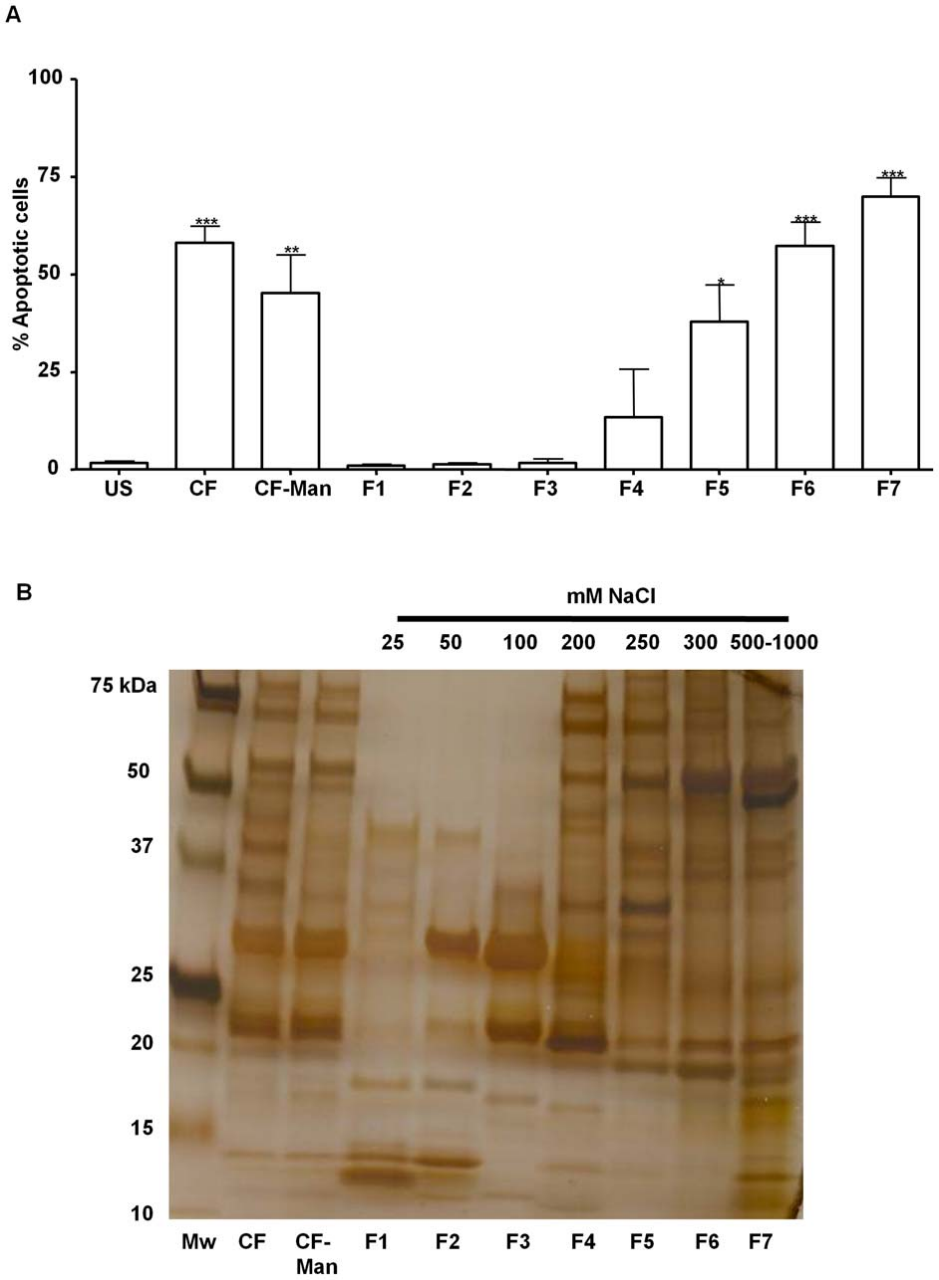
Separation of CF by ConA affinity chromatography and DEAE-Sepharose chromatography yielded a fraction with potent apoptotic activity in human monocytes. *A.* Apoptosis induced by DEAE-Sepharose fractions 1 to 7 (F1 to F7) and unfractionated CF was measured by flow cytometry and presented as percentage of human monocytes that stained annexinV positive *** significance p<0.001, ** significance p<0.01, * significance p<0.05 as compared to unstimulated control. *B.* SDS-PAGE and silver staining of fractions of CF after ConA affinity (CF-Man) and DEAE-Sepharose chromatography (F1 to F7). Mw denotes the molecular mass standards.

Infection of human derived monocytes for 48 h with live, virulent *M. tuberculosis* (MOI of 5∶1) induces early apoptotic events that are defined by annexinV positive and propidium iodide negative staining [12]. However, apoptosis does not occur with formaldehyde-fixed or heat-killed *M. tuberculosis*. Thus, it was hypothesized that induction of apoptosis in human monocytes was due to a secreted product of *M. tuberculosis* [11]. Towards purifying the responsible mycobacterial product(s), the CF from *in vitro* cultures of *M. tuberculosis* H37Rv was shown to induce monocyte cell membrane damage similar to the live infection (Fig. 1A) [12]. As a first step in the purification scheme the ManLAM, previously shown to inhibit macrophage apoptosis [15], [17], [18], was removed by ConcanavalinA-affinity chromatography to give Man-LAM depleted CF (CF-Man). This material was further resolved on DEAE-Sephadex with a stepwise gradient of sodium chloride from 0–1000 mM, resulting in seven fractions (Fig. 1B). Evaluation of each fraction for induction of apoptosis via annexinV staining, demonstrated that fraction 7 induced the strongest response with nearly 70% of human monocytes displaying early cell membrane damage (Fig. 1A) (a small percentage of cells was dually labeled with annexinV and propidium iodide, data not shown). Removal of ManLAM by ConA chromatography consistently reduced the biological activity of CF (Fig. 1A), but this reduction was not statistically significant (p>0.05). In addition to binding ManLAM, ConA chromatography could also be retaining mannosylated molecules such as LM and the 19 kDa lipoprotein, which are known to induce apoptosis [19], [20], [25]. Considerable activity was also associated with fraction 5 and 6. However, further purification and characterization was targeted to fraction 7 as it was the most potent.

**Figure 2:**
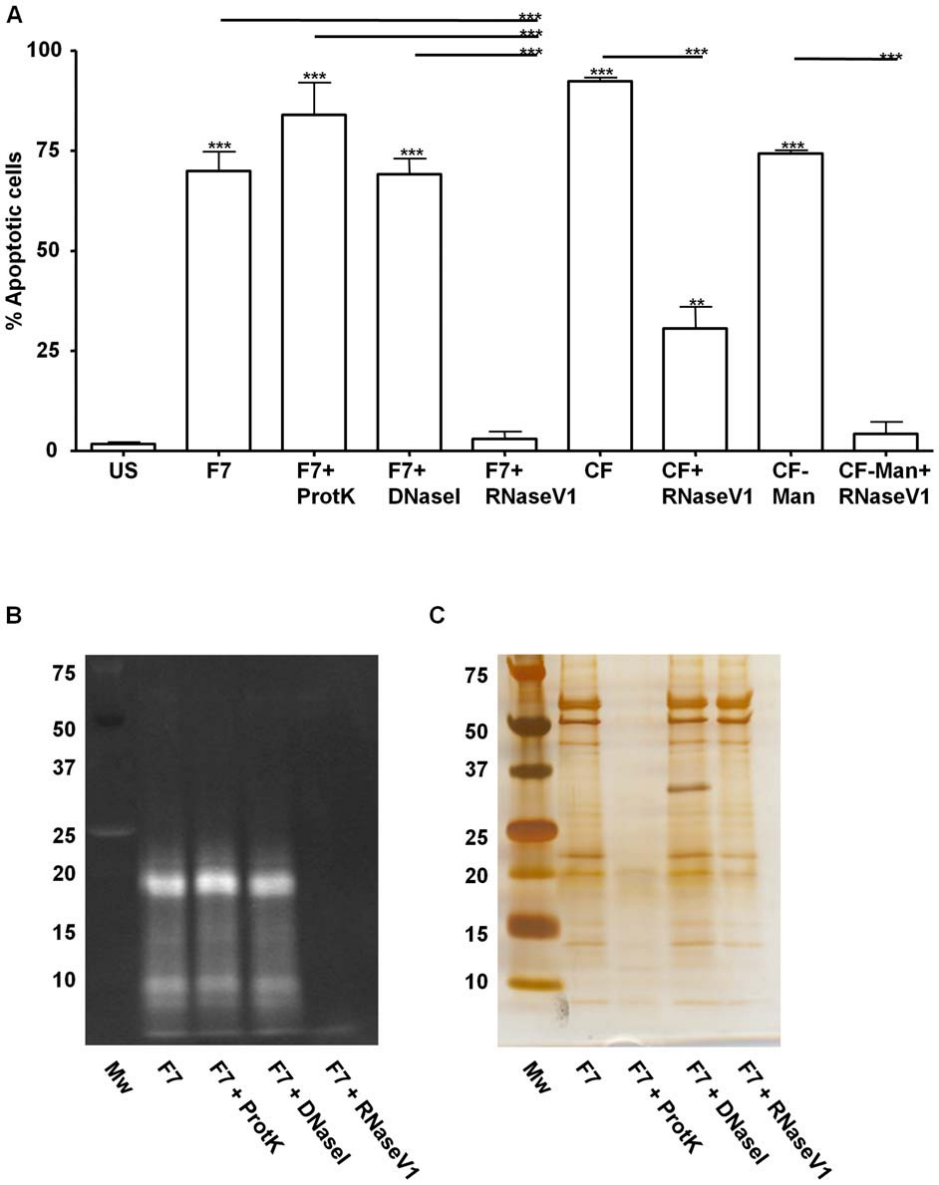
RNA in DEAE-Sepharose Fraction 7 induces apoptosis in human monocytes. *A.* Apoptosis induced by DEAE-Sepharose Fraction 7 (F7), F7 treated with proteinase K (F7+ProtK), F7 treated with DNase1 (F7+DNase1), F7 treated with RNaseV1 (F7+RNaseV1), CF, CF treated with RNaseV1 (CF+RNaseV1), CF-Man, CF-Man treated with RNaseV1 (CF-Man+RNaseV1). Apoptosis was measured by flow cytometry and presented as percentage of human monocytes that stained annexinV positive. *** significance p<0.001, ** significance p<0.01 as compared to unstimulated control or between treated samples. *B.* SDS-PAGE with ethidum bromide staining and *C.* silver staining of F7 and F7 treated with proteinase K, DNase1, or RNaseV1.

The chemical nature of the apoptotic-inducing mycobacterial molecule(s) present in fraction 7 was initially determined by assessing the loss of stimulation after differential enzymatic digestion with Proteinase K, DNaseI, or RNaseV1 (Fig. 2A). Unexpectedly, the apoptosis-inducing activity of fraction 7 was completely abrogated by treatment with RNaseV1, as there was a 95% reduction in the number of annexinV positive cells. Digestion with DNaseI had no impact, whereas Proteinase K treatment increased the number of monocytes in early apoptosis (80% of monocytes), but was not statistically significant. Treatment of monocytes with enzymes alone had a negligible effect on apoptosis (data not shown). Evaluation of fraction 7 by SDS-PAGE and ethidium bromide or silver staining pre and post enzymatic digestions (Fig. 2B and C) confirmed the presence of nucleic acids in fraction 7. These nucleic acids migrated at <20 kDa by SDS-PAGE and were only eliminated by digestion with RNaseV1 (Fig. 2B). It was also noted that Proteinase K, but not RNaseV1 or DNaseI treatment resulted in the elimination of proteins present in fraction 7. Furthermore, RNaseV1 treatment was additionally performed on CF and CF-Man to determine the RNA's contribution to monocyte cell membrane damage induced by these complex fractions. As determined by evaluating the samples pre- and post-RNaseV1 digestion, the RNA was responsible for almost 70 and 90% of the biological activity from CF and CF-Man, respectively (Fig. 2A). Incubation with 1 µg/ml of CF would contain approximately 30 ng of RNA, (Sheldon and Belisle, unpublished results), highlighting the biological potency of this product.

### Definition of Biologically Active RNA

**Figure 3:**
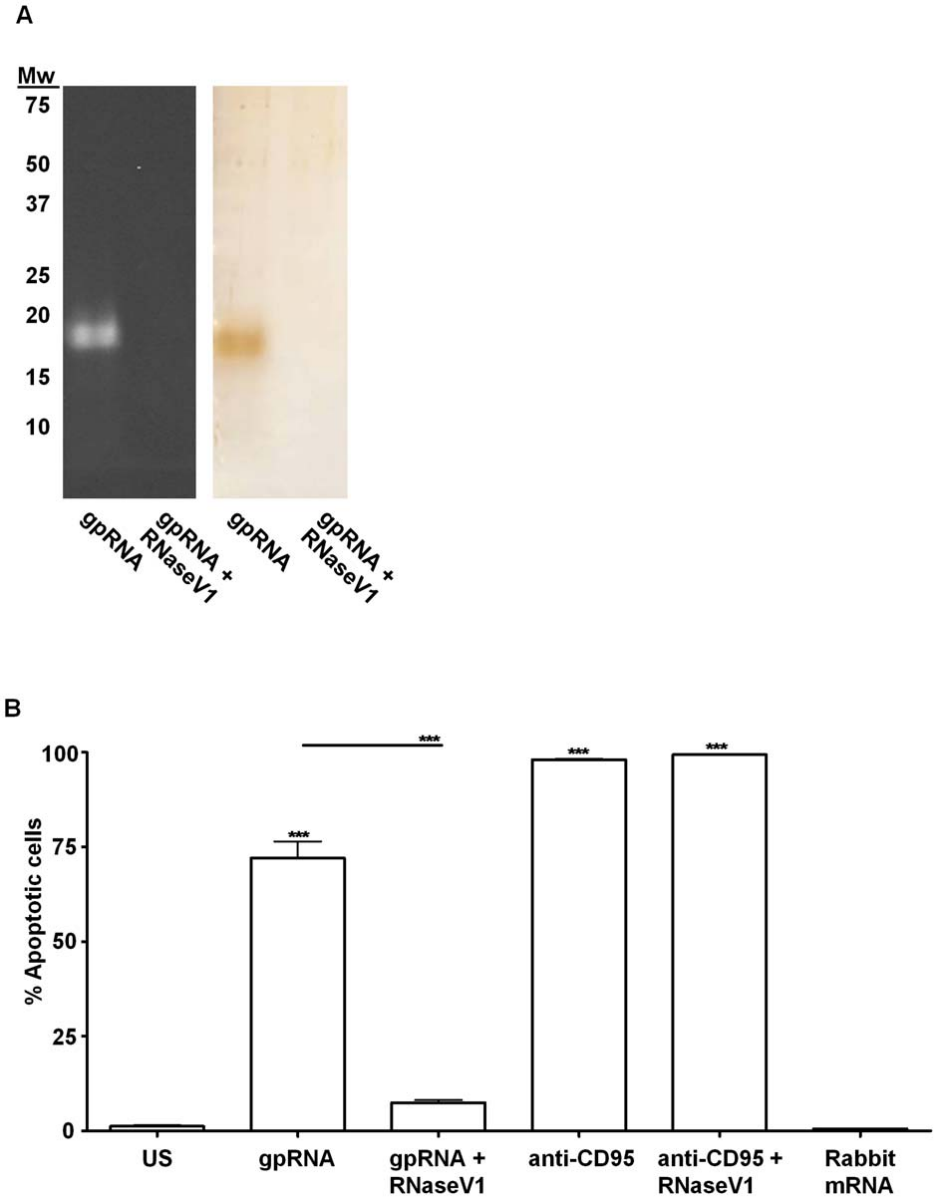
Human monocyte apoptosis is specifically induced by gel-purified mycobacterial RNA. *A.* SDS-PAGE with ethidium bromide (left) and silver stained (right) gel-purified RNA (gpRNA) from F7 and treated with RNaseV1 (gpRNA+RNaseV1). *B.* Monocyte apoptosis induced by gpRNA untreated or treated with RNaseV1, anti-CD95 untreated or treated with RNaseV1 (anti-CD95+RNaseV1) and rabbit mRNA. Significance as described above.

To confirm that RNA was responsible for the biological activity, fraction 7 was digested with DNaseI and Proteinase K, extracted with phenol/chloroform/isoamyl alcohol (25∶24∶1) to remove contaminating lipids and undigested proteins and the resulting RNA was gel purified from a denaturing urea-polyacrylamide gel (Fig. 3A). Gel purified RNA was subjected to RNaseV1 treatment and compared to untreated RNA in the biological assay with human monocytes. The apototic activity of the gel purified RNA was retained after purification (72% apoptotic monocytes), and this activity was abrogated by RNaseV1 treatment (7.5% apoptotic monocytes) (Fig. 3A and B). As a control to ensure RNaseV1 was not modulating monocyte apoptosis via a different mechanism, we evaluated cell membrane damage induced by the well characterized anti-CD95 pathway in the presence or absence of RNaseV1. No difference in AnnexinV positive cells was observed when monocytes were co-incubated with anti-CD95 and RNaseV1 versus anti-CD95 alone (Fig. 3B). Finally, to evaluate whether monocyte cell membrane damage could be induced by other sources of exogenous RNA, monocytes were incubated with rabbit mRNA; no induction of monocyte apoptosis was observed (Fig. 3B). These results confirmed that fraction 7 obtained from *M. tuberculosis* H37Rv CF is enriched in RNA, which was the sole inducer of human monocytes cell membrane damage.

To further define the RNA present in the CF the same strategy utilized to clone siRNA was adopted [26], [27]. From a library of cDNA clones plasmids were purified from a total of 33 clones. Sequence analyses of the plasmid inserts revealed that the *M. tuberculosis* RNA present in the CF predominantly consisted of tRNA and rRNA with lengths between 30 to 70 bases (Table 1). Of the 17 tRNA sequences, a majority corresponded to tRNA^Asp^ fragments and a total of 15 clones represented 16S or 23S rRNA fragments, indicating selectivity in the RNA species released into the CF. Sequence alignments for both the 16S and 23S rRNA showed that the majority of fragments originated from different regions of the mature rRNA and only occasionally did two fragments correspond to the same region (Fig. S1, upper and middle panel). Alignment of the cloned tRNA fragments determined that they were truncated at both the 5′ and 3′ ends (Fig. S1, lower panel).

**Table 1:** Sequence analysis of cloned extracellular mycobacterial RNA fragments present in *M. tuberculosis* CF.

Cloned RNA	Number of Clones 2
tRNA^Asp^ 1	10
tRNA^Asn^	4
tRNA^Lys^	1
tRNA^Thr^	2
23S rRNA^1^	9
16S rRNA^1^	6
mRNA	1

1 The specific regions of the RNA sequences represented by each clone are depicted in Figure S1.

2 The number of clones represents the total number of sequences observed for each RNA species.

### Kinetics of RNA Release

As tRNA and rRNA are highly abundant intracellular constituents, kinetic studies were performed to evaluate when the *M. tuberculosis* RNA started to accumulate in the CF and if its presence was attributable to the CF being harvested from late logarithmic (14 day), presumably autolytic, cultures. After extensively washing cells with GAS medium, CF was collected at different time points (0 to 28 days) and analyzed by SDS-PAGE and ethidium bromide staining. Interestingly, the presence of *M. tuberculosis* RNA paralleled *M. tuberculosis* protein secretion into the CF. Specifically, the RNA began to accumulate between two and three days of culture (early log phase), becoming more abundant as the culture progressed (Fig. S2). Similar results were obtained when the CF was immediately precipitated with acetone after harvesting, to rule out the possibility that higher molecular weight RNA species were being degraded to 30 to 70 bases during the protracted processing of CF (not shown). These results suggest that in addition to secreted proteins, *M. tuberculosis* H37Rv actively releases rRNA and tRNAs into the CF and these accumulate as stable products starting in early log phase.

### Mechanism of RNA Induced Apoptosis

**Figure 4:**
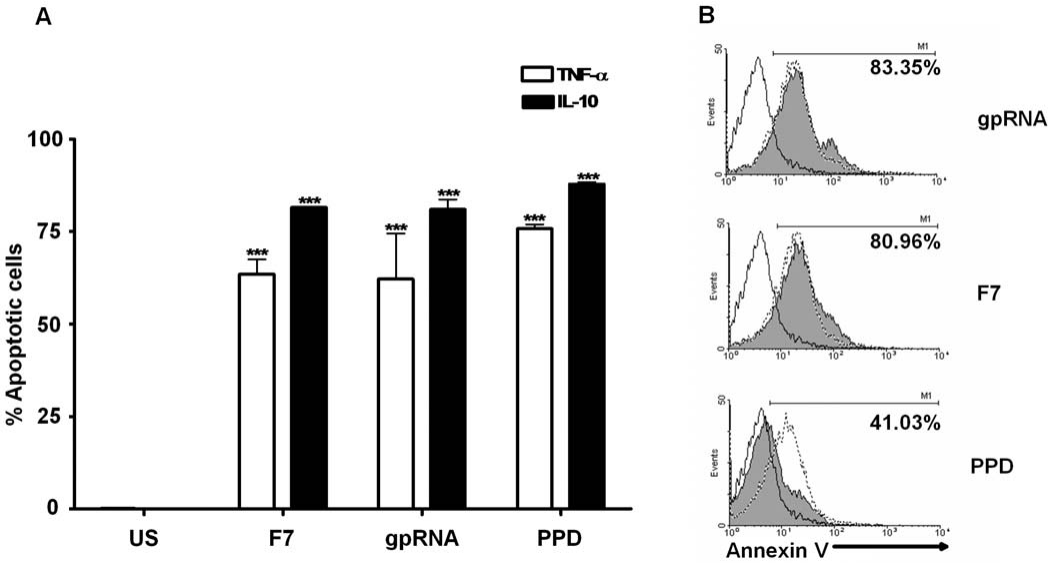
Human monocyte apoptosis induced by extracellular *M. tuberculosis* H37Rv RNA is TNF-α independent. *A.* TNF-α (open bars) and IL-10 (closed bars) induced in human monocytes treated with DEAE-Sepharose Fractions 7 (F7), gel purified *M. tuberculosis* H37Rv (gpRNA) and purified protein derivative (PPD) from *M. tuberculosis* were measured by flow cytometry and the data presented as the percentage of human monocytes producing the cytokine, *** significance p<0.001 as compared to unstimulated control. *B.* Human monocytes were incubated with gpRNA, F7 or PPD, and annexinV staining was measured by flow cytometry (open histogram with dotted line) to assess apoptosis. Treatment of monocytes with anti-TNF-α for 1 h prior to stimulation with F7, gpRNA, or PPD demonstrated no alteration in annexinV staining for monocytes incubated with F7 or gpRNA, but did result in decreased annexinV staining for monocytes incubated with PPD (closed histogram). The open histograms with a solid line correspond to unstimulated monocytes.

In order to assess the mechanism by which RNA induced apoptosis, TNF-α and IL-10 production was evaluated since the balance between these two cytokines is a factor in modulating cell death [12]–[14], [16]. *M. tuberculosis* RNA induced the intracellular production of these two cytokines to a level similar to that induced by the positive control, PPD (Fig. 4A). However, in contrast to PPD, the cell membrane alterations induced by RNA were not blocked with anti-TNF-α (Fig. 4B).

**Figure 5:**
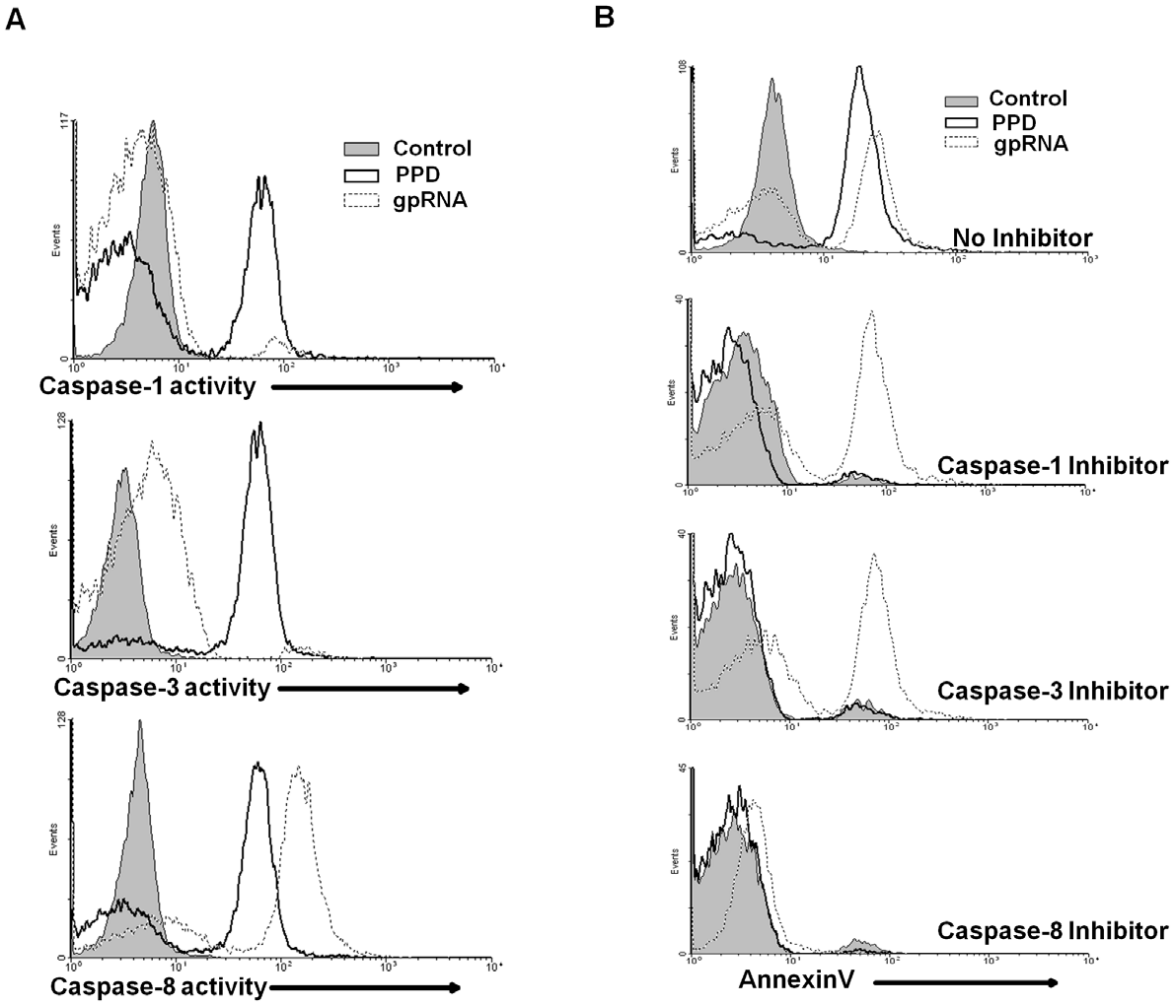
The *M. tuberculosis* RNA's activity is caspase-8 dependent. *A.* Flow cytometry demonstrated *M. tuberculosis* H37Rv RNA (gpRNA) activated caspase-8. Human monocytes were stimulated with 1 µg/ml of purified *M. tuberculosis* H37Rv RNA gpRNA (open histogram with dotted line) or PPD (open histograms with solid line) for 48 h and caspase activity was determined by flow cytometry after staining with FLICA-YVAD-FMK (caspase-1), FLICA-DEVD-FMK (caspase-3), or FLICA-LETD-FMK (caspase-8). Tissue culture medium without RNA or PPD was used as the control (closed histogram). *B.* Stimulation of apoptosis based on annexinV staining was measured for monocytes stimulated with gpRNA or PPD (top histogram) and when the monocytes were pretreated for 1 h with 10 nM caspase-1 (YVAD-FMK), caspase-3 (DEVD-FMK), or caspase-8 (LETD-FMK) inhibitors (lower histograms).

Given the importance of caspase activity in TNF-α dependent and independent apoptotic pathways [1], [4], and recent reports that bacterial RNA activates caspase-1 [28], experiments were performed to determine whether *M. tuberculosis* H37Rv RNA activates caspases. *M. tuberculosis* RNA strongly activated caspase-8 (LETD-FMK), intermediately activated caspase-3 (DEVD-FMK) and had negligible effect on caspase-1 (YVAD-FMK), as determined by flow cytometry (Fig. 5A). In contrast, PPD activated caspase-1, 3 and 8 to similar extents. Consistent with these data, only caspase-8 inhibitor abrogated the RNA induced cell membrane alterations, whereas all the caspase inhibitors significantly abrogated PPD's activity (Fig. 5B). These findings provide evidence that *M. tuberculosis* RNA activates apoptosis via a caspase-8 signaling mechanism and that this route of activation differs from those observed for other mycobacterial products [12].

**Figure 6:**
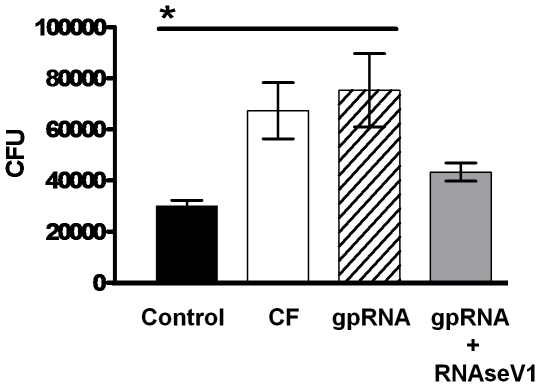
*M. tuberculosis* H37Rv RNA altered human monocyte's ability to control *M. tuberculosis* infection. CFUs were determined for human monocytes infected with *M. tuberculosis* H37Rv and incubated for four days in the presence of 1 µg/ml of *M. tuberculosis* H37Rv CF, purified RNA (gpRNA), or purified RNA digested with RNaseV1 (gpRNA+RNaseV1). The presence of CF or gpRNA resulted in a significant increase in CFUs as compared to the untreated infected monocytes (Control). Data represent the mean ± SEM of 3 replicates of the same experiment (*p<0.03).

Lastly, the ability of RNA to alter monocyte's control of mycobacterial growth was examined by treating *M. tuberculosis* infected human monocytes with purified RNA. Determination of colony forming units (CFUs) after four days of incubation revealed a greater than two fold increase in the number of bacilli associated with RNA treated vs. untreated monocyte cultures (Fig. 6). Furthermore, digestion with RNaseV1 abrogated the RNA's deleterious effect on monocyte control.

## Discussion

Understanding apoptosis at a molecular level is critical to elucidating the pathogenesis of tuberculosis. Recent *in vivo* evidence suggests that apoptosis is an active mechanism in necrotic granulomas [8], a pathological hallmark of tuberculosis that is widely accepted as being key to the bacterium's persistence and transmission [29]. Our search for a *M. tuberculosis* product that replicated the apoptotic activity observed with a live *M. tuberculosis* infection [11], led to the surprising discoveries firstly that low molecular weight RNA fragments were secreted into the CF, long known as a source of major *M. tuberculosis* protein antigens, and secondly that these RNA fragments were major apoptotic factors. The role of mycobacterial RNA in the pathogenesis of tuberculosis was initially proposed by Youmans and colleagues in the 1960s and 70s [30], [31]. In their studies, ribosomal RNA obtained from whole cells increased bacterial burden when introduced into infected animals. Our experiments demonstrated that apoptosis induced by RNA significantly altered the ability of the monocytes to control *M. tuberculosis* growth. Although these studies did not assess *in vivo* growth of *M. tuberculosis* in a manner similar to Youmans and colleagues, they do provide support and a potential explanation for the early observations.

The activation of specific caspases is central to the induction of apoptosis and classically differentiates between the mitochondrial and apical pathways [1], [5]. The strong induction of caspase-8 activity with the *M. tuberculosis* RNA fragments and the prevention of apoptosis with a caspase-8 inhibitor provided convincing evidence for the induction of apoptosis via the apical pathway. The *M. tuberculosis* RNA fragments also stimulated the production of TNF-α and although TNF-α is primarily recognized as a mediator of inflammation, it can also induce apoptosis via caspase-8 when it binds to the TNF-R1 [4], [5]. However, anti-TNF-α antibodies did not inhibit the induction of apoptosis by *M. tuberculosis* RNA fragments. Similar to what has been reported during macrophage infection with virulent *M. tuberculosis* [32], it is possible that mycobacterial RNA induces TNF-R2 shedding thus rendering monocytes unresponsive to TNF-α. Our previous work as well as that of others also indicated a potential role for caspase-1 in *M. tuberculosis* mediated apoptosis [14], [15]; however, the current studies demonstrated that *M. tuberculosis* RNA induction of apoptosis was caspase-1 independent. This lack of caspase-1 involvement also suggests that the *M. tuberculosis* RNA is not inducing an inflammasome response [33]. We did not, however, investigate whether the RNA fragments inhibited caspase-1 activity as has been recently reported to occur with *M. tuberculosis* infections [34]. In the apical pathway of apoptosis, caspase-3 is defined as the effector caspase and is downstream of caspase-8 [1], [5]. Our studies unexpectedly revealed that treatment of monocytes with *M. tuberculosis* RNA fragments only resulted in a modest increase in caspase-3 activity, as compared to that induced by PPD, and inhibition of caspase-3 failed to prevent *M. tuberculosis* RNA induced apoptosis. Thus, while the caspase-8 data suggest that the excreted *M. tuberculosis* RNA fragments stimulate monocyte apoptosis via the apical pathway, the non-essentiality of TNF-α or caspase-3 indicated an alternative mechanism of induction. One potential mechanism is that described for the “type II” apoptotic cells where capase-8 activation directly induces a mitochondrial pathway of apoptosis and is associated with FAS or TNF-related apoptosis-inducing ligand (TRAIL) mediated signaling [5]. Our studies provide additional experimental evidence to the increasing observations that caspase-8 plays a critical role not only during *in vitro* experiments with mycobacteria-infected macrophages but also in patients with active disease [35], [36]. Future work will assess the specific apoptotic pathway and signaling events stimulated by excreted *M. tuberculosis* RNA.

The RNA fragments characterized in this study possess numerous dsRNA hairpin structures, and studies with other viral and bacterial dsRNAs demonstrate that interactions with TLR-8 or cryopyrin led to caspase activation [28], [37], [38]. Interactions between dsRNA and TLR-8 result in caspase-8 activity [37], [38] and TLR-8 polymorphisms are associated with increased susceptibility to tuberculosis [39]. Thus, activation of host monocytes via TLR8 would fit the data presented for the *M. tuberculosis* RNA fragments. In contrast, bacterial RNA interactions with cryopyrin activate caspase-1 [28], an event that did not occur with the *M. tuberculosis* RNA fragments. It must also be considered that tRNA and rRNA undergo multiple species-specific modifications [40], [41] that could alter their ability to activate different pattern recognition receptors (PRRs) and signaling pathways. In contrast, mammalian mRNA, which is known to possess different modifications [41], did not activate monocyte apoptosis as the mycobacterial RNA fragments. Therefore, other PRRs for the *M. tuberculosis* RNA fragments need to be considered in future studies such as Protein Kinase R (PKR) that is activated in macrophages infected with *M. bovis* BCG [42], the endosomal TLR-3 and TLR-7 [43], [44], and cytoplasmic receptors Rig-1, MDA-5, DAI and Nod [45]. The stability of the *M. tuberculosis* RNA fragments must also be considered with respect to accumulation within the host. The complex secondary structure of the tRNA and rRNA might protect against host as well as bacterial RNase activity, and allow for its stable accumulation in the granuloma.

Multiple classes of pathogen derived macromolecules are defined as modulators of innate immunity, including RNA and DNA [46]. However bacterial nucleic acids typically are not described as being extracellular products. Thus, the isolation of the small RNA fragments from the CF of *M. tuberculosis* as the primary apoptotic factor was unexpected. The possibility that release of RNA fragments is due to lysis of *M. tuberculosis* was investigated by following the kinetics of RNA release. Such analyses revealed that accumulation of the secreted RNA fragments paralleled the kinetics of *M. tuberculosis* protein secretion. RNA was observed in the CF as early as 2 days, the same point in time used to distinguish truly secreted proteins from those resulting from autolysis [47]. Additionally, there did not appear to be a massive accumulation of extracellular RNA during late log-phase that would imply cell lysis as the underlying mechanism. Although a specific mechanism of active transport of the RNA fragments was not identified, there are suggestions by others that the ESX (Type VII) secretion apparatus of *Mycobacterium* spp. is involved in DNA transfer or conjugation [48]. There are multiple ESX like secretion modules encoded on the *M. tuberculosis* genome [49] and investigation into whether one or more of these is involved in secretion of the RNA fragments is on-going. Our current data does not completely rule out that early bacterial lysis or cell division contributed to RNA release. It is well known that other intracellular molecules such as heat shock proteins strongly activate the host immune system during *in vivo* mycobacterial infections [50], [51]. It is possible that early bacterial lysis allows for the release of significant quantities of mycobacterial proteins and RNA in host granulomas where it could induce host cell apoptosis leading to necrotic granulomas, as well as modulate granuloma environment in favor of bacterial survival. In this context, it was interesting to observe that mycobacterial RNA fragments affected the monocyte's ability to control infection despite stimulating TNF-α production. TNF-α production is required for both granuloma formation and bacterial control but alone is not sufficient [29]. The anti-inflammatory cytokine IL-10 which was also induced by the RNA fragments could be counteracting TNF-α. Alternatively, it was recently observed that intranasal delivery of the synthetic RNA poly-IC, increased bacterial counts in tuberculosis infected animals by stimulating type-I IFNs [52]. Even though we did not evaluate this family of cytokines, we obtained similar enhanced monocyte “permissiveness” to *M. tuberculosis* infection when stimulating with mycobacterial instead of a synthetic RNA.

Multiple studies have evaluated how different types of macrophage cell death affect mycobacterial survival [11], [12], [53]–[56]. In general, the current dogma is that macrophage apoptosis in contrast to necrosis, is an innate mechanism to control mycobacterial infection [55]. Consistent with this, apoptotic macrophages controlled growth of avirulent mycobacteria, whereas it was unrestricted for virulent mycobacteria in necrotic macrophages [57]. Hence, macrophage necrosis was proposed as a pathogenesis mechanism of virulent mycobacteria [57]. Alternatively, recent mechanistic studies have suggested that inhibition of apoptosis completion might actually be a cause for mycobacterial-induced macrophage necrosis [55]. Mitochondrial depolarization, PS exposure and membrane microdisruptions occurring early during cell death were similarly observed in macrophages finally succumbing to necrosis or apoptosis [57]–[59]. The critical events tipping the balance to necrosis or apoptosis, as well as bacterial survival take place further downstream during cell death and appear to be modulated by the antagonizing effects of prostaglandin E2 and lipoxin [57]–[61]. Similarly, in our studies we identified that mycobacterial RNA activated early events in monocyte apoptosis such as PS exposure, TNF-α production and activation of the initiator caspase-8. In contrast, activation of the effector caspase-3 was not significant and actually was not involved in PS exposure. Thus, it remains to be determined whether mycobacterial RNA inhibits the downstream completion of apoptosis, explaining enhanced mycobacterial growth in infected monocytes exposed to this product. It must also be considered that our experiments were performed with freshly isolated human monocytes, whereas most of the other studies have been performed with fully differentiated macrophages. As recently reported by some members of our group [62], human monocyte phenotype and susceptibility to mycobacterial-induced cell death is significantly altered during the differentiation process. Finally, the effect of mycobacterial RNA could also be synergized by additional secreted mycobacterial molecules in the macrophage phagosome [63] or alternatively, as non-differentiated monocytes migrate to necrotic granulomas harboring extracellular mycobacteria.

A hallmark of *M. tuberculosis* is the bacterium's ability to interact with the host immune response in a manner that allows for its own survival and at the same time causes active disease in only a relatively small percentage of the individual it infects. With this perspective it is not surprising that this pathogen has evolved a means to take advantage of the eukaryotic mechanism of programmed cell death. Likewise, the involvement in this process of a bacterial product that is released or secreted only during active growth is not surprising. Thus, the continued elucidation of the exact signaling mechanisms exploited by *M. tuberculosis* to induce apoptosis as well as the process the bacterium utilizes to release RNA fragments will allow further definition of tuberculosis pathogenesis and the biology of this highly successful pathogen.

## Materials and Methods

### Growth of M. tuberculosis

To prepare bacilli for monocyte infections, *M. tuberculosis* H37Rv (kindly provided by Laboratorio de Micobacterias, Instituto Nacional de Salud, Bogota, Colombia) was grown as a pellicle in Middlebrook 7H9 medium (Becton Dickinson, Sparks, MD) supplemented with glycerol (Promega, Madison, WI), oleic acid-albumin-dextrose-catalase (OADC) (Becton Dickinson), and Tween 80 (Becton Dickinson). Cultures were collected and *M. tuberculosis* cells washed twice with phosphate buffered saline (PBS). To disrupt bacterial clumps, *M. tuberculosis* was suspended in RPMI-1640 with 20% glycerol and probe sonicated (Model CV33 Sonics Vibra Cell, Newtown, CT) five times at 2.5 W output for 1 min at 4°C. The cell suspensions were stored at −70°C until use. CFUs were determined prior to monocyte infections by plating 100 µL of serial dilutions of an *M. tuberculosis* stock on Middlebrook 7H10 agar (Becton Dickinson) supplemented with glycerol and OADC. Colonies were counted after two weeks of incubation at 37°C.

### Isolation of Extracellular RNA

For isolation of biologically active fractions, *M. tuberculosis* strain H37Rv was grown in glycerol alanine salts (GAS) medium under constant rotation in roller bottles for two weeks at 37°C and culture filtrate (CF) was obtained as previously described [64]. Lyophilized CF was suspended overnight at 4°C at 1 mg/ml protein concentration in ConcanavalinA (ConA) binding buffer [65], and passed over a ConA-Sephadex column (Sigma-Aldrich, St. Louis, MO) to remove mannosylated glycoproteins and lipoglycans (LM and LAM). The flow-through fraction was collected, concentrated, and washed three times with 20 mM Tris (pH 8.1). The CF depleted of mannosylated glycoconjugates (25 mg) was applied to a 1×10 cm column packed with 5 ml of DEAE-Sepharose (Amersham Biosciences Corporation, Piscataway, NJ) equilibrated with Tris 20 mM (pH 8.1). The column was washed with 20 mM Tris 20 (pH 8.1) and eluted with stepwise increases (25 to 1000 mM) of NaCl in 20 mM Tris (pH 8.1). Each fraction was concentrated and dialyzed against 10 mM ammonium bicarbonate, filtered sterilized, lyophilized and stored at −20°C until used.

To prepare highly purified RNA, the material eluted from DEAE-sepharose with 500–1000 mM NaCl was suspended in 0.1 ml of 10 mM ammonium bicarbonate, 1 mM MgCl_2_ to a final concentration of 0.4 mg/ml and digested overnight at 37°C with RNase-free DNaseI (Sigma-Aldrich), followed by RNase-free Proteinase K (Sigma-Aldrich) for 8 h at 37°C at a final concentrations of 0.04 mg/ml. The digested material was partitioned with an equal vol of phenol∶chloroform∶isoamyl alcohol (25∶24∶1) followed by an equal vol of chloroform∶isoamyl alcohol (24∶1). RNA was precipitated from the final aqueous phase with 0.1 vol of 3 M sodium acetate (pH 5.3) and 2.5 vol of ethanol, and collected by centrifugation (13,000× g, 30 min). Final purification was performed by preparative gel electrophoresis using a denaturing urea poly-acrylamide gel [26]. RNA preparations were suspended in PBS, and filter sterilized. The gel purified RNA was incubated overnight at 37° C with or without 0.1 U/µl RNaseV1 (Ambion, Austin, TX) and lyophilized. Endotoxin contamination was determined using the Lymulus Amebocyte Lysate assay (Bio-Whittaker, Walkersville, Maryland). RNA concentration was determined by densitometry using Quantity One software (Version 4.1.1 Bio-Rad, Hercules, CA) after resolving the *M. tuberculosis* RNA and standards by denaturing urea poly-acrylamide gel electrophoresis and staining with ethidium bromide. To assess for the presence of LAM, samples (1 µg) were subjected to Western blot analysis using the LAM specific monoclonal antibody CS-35 as the probe [66].

To evaluate the kinetics of mycobacterial RNA release into the CF, low passage *M. tuberculosis* H37Rv was centrifuged at 3,000 rpm and washed three times with medium. Bacterial pellets were suspended in GAS medium and used to inoculate 400 ml cultures. CF was collected at different time points of incubation and processed. Alternatively, CF was precipitated overnight at −20°C with three volumes of ice cold acetone. The precipitated material was collected by centrifugation and analyzed by SDS-PAGE [67].

### RNA cloning and sequencing

Gel purified RNA was cloned as described by Elbashir and Lau for small interfering (si)RNA [26], [27]. Briefly, the RNA was dephosphorylated and ligated to the 3′adaptor [5′ phosphorylated uuu AAC CGC ATC CTT CTC iT-3′; lower case letters ribonucleotides, capitalized letters deoxyribonucleotides, (Dharmacon, Lafayette, CO)]. The ligation products were gel purified, phosphorylated at the 5′ end with T4 polynucleotide kinase (New England Biolabs, Beverly MA) and ligated to the 5′adaptor sequence [5′ TAC TAA TAC GAC TCA CT aaa 3′ (Dharmacon)]. Synthesis of cDNA from adaptor-ligated RNA was accomplished using the Thermoscript kit (Invitrogen) and the reverse primer (5′ GAC TAG CTG GAA TTC AAG GAT GCG GTT AAA-3′). Double stranded DNA was generated by PCR amplification with the reverse primer, and the forward primer (5′CAG CCA ACG GAA TTC ATA CGA CTC ACT AAA-3′). An aliquot of the PCR products was digested with *EcoR*I (New England Biolabs) and ligated to form concatemers that were resolved by agarose gel electrophoresis and extracted. Overhangs of poly-A were added with Taq Platinum polymerase in the presence of dNTPs. The DNA fragments were ligated with pCR 2.1 TOPO vector (Invitrogen), transformed into TOP10 *E. coli* (Invitrogen) and individual clones grown for plasmid isolation. The cloned DNA fragments were sequenced through Macromolecular Resources Facility (Colorado State University). Plasmid sequences were analyzed with the Vector NTI software (BioExchange, San Francisco, CA) to identify the 5′ and 3′ adaptor sequences, and the intervening regions were searched against the *M. tuberculosis* genome, via BLAST analysis.

### Monocyte infection and stimulation

Venous blood (120 ml) was obtained from healthy volunteers after signing an informed consent form describing the protocol for human subjects, which was approved by the Ethics Committee of the Facultad de Medicina, Universidad de Antioquia. The blood was defibrinated by continuous agitation with glass beads, centrifuged, and the buffy coat suspended in 3 vol of PBS. Mononuclear cells were separated on Histopaque, density 1.077 g/ml (Sigma-Aldrich) by centrifugation at 400× *g*, and cell viability determined by trypan blue exclusion. Cells were suspended at 1×10^6^ cells/ml in RPMI-1640 medium (Gibco BRL) without antibiotics and supplemented with 0.5% heat-inactivated autologous serum (AS). The ratio of monocytes was determined by flow cytometry with anti–CD14-PE staining (clone M5E2, Becton Dickinson-Pharmingen). CD14+ cells (2×10^5^/well) were plated in 48-well flat-bottomed culture plates (Corning, Corning, NY) for 4 h at 37°C, and non-adherent cells removed by repeatedly washing with prewarmed PBS with 0.5% AS. RPMI-1640 supplemented with 10% AS (1 ml) was added to each well. The number of adherent cells was determined by mechanical removal and counting in a haemocytometer.

Adhered monocytes were infected with viable *M. tuberculosis* at a multiplicity of infection of 5∶1 for 4 h. Extracellular bacilli were removed by repeatedly washing with prewarmed PBS. RPMI-1640 supplemented with 10% AS (1 ml) was added to each well. The CF and RNA preparations were added at 1 µg/ml protein or nucleic acid concentration, respectively. Cultures were incubated at 37°C, 5% CO_2_ for 96 h, and the number of viable bacilli determined by plate counting after lysis of host cells with water containing 0.1% saponin [12].

For analysis of apoptosis induction or cytokine production the CF, individual CF fractions, and purified mycobacterial RNA or rabbit mRNA (Sigma) were added at 1 µg/ml protein or nucleic acid concentration, respectively. PPD was used as a control at 10 µg/ml. As a control, induction of human monocytes apoptosis was performed by incubating 10^6^ cells with 2 µg/ml of anti-human CD95 (BD Pharmingen).

### Determination of intracellular TNFα and IL-10 by flow cytometry

Evaluation of intracellular cytokines was based on previous methods [14]. Briefly, cells were cultured for 18 h with mycobacterial products at 37°C and 5% CO_2_. Brefeldin A (1 µg/ml) was subsequently added and the cultures were incubated for 6 h. Cells were washed twice with PBS, fixed with 2% paraformaldehyde in 0.1 M NaH_2_PO_4_ for 20 min at room temperature, and harvested. Intracellular cytokine labeling was achieved by washing the cells once with permeabilization buffer [PBS (pH 7.4) containing 1% PHS, 1% BSA, 0.1% sodium azide and 0.1% saponin], and incubating in 200 µl of permeabilization buffer containing 2.5 µg mAb anti-TNF-α-FITC and 2.5 µg anti-IL-10-PE, or IgG2a-FITC and IgG2b-PE as isotype controls for 30 min at 4°C. Cells were washed three times with PBS containing 1% PHS, 1% BSA, and 0.1% sodium azide, pH 7.4. As control for the intracellular signal, non-permeabilized cells were stained with the TNF-α and IL-10 specific antibodies. Thereafter cells were analyzed by flow cytometry with a FACS EPICS XL (Coulter, Hialeah, FL). The percentage of positive cells and the mean fluorescence intensity were determined using Windows Multiple Document Interface 2,8 software (WinMDI, Scrips Research Institute, La Jolla, CA).

### Determination of cell death by flow cytometry using annexinV and propidium iodide

Cells were cultured for 48 h in the presence of the mycobacterial products and incubated for 30 min at RT in darkness with 5 µl of FITC-labeled annexin-V (Molecular Probes, Invitrogen), 10 µl of propidium iodide at 1 µg/ml (ICN Biomedicals, Costa Mesa, CA), and 100 µl of PBS. Cells were washed with PBS and analyzed by FACS [11].

To assess whether apoptosis was dependent on TNF-α signalling, monocytes were incubated for 1 h with purified anti–TNF-α (MAb11, Becton Dickinson-Pharmingen), followed by the addition of the different stimuli for 36 h. Thereafter, annexinV staining was evaluated.

### Caspase activation

Caspase activation was measured at 48 h post addition of *M. tuberculosis* products to the adhered monocytes. The activation of caspase-1, 3, and 8 was determined by flow cytometry as previously described [12]. To evaluate whether caspase activation was required for apoptosis, monocytes were incubated with 10 nM of caspase-1 (YVAD.fmk), caspase-3 (DEVD.fmk), or caspase-8 (IEMD.fmk) specific inhibitors (Sigma-Aldrich) for 1 h prior to the addition of mycobacterial products. Dimethylsulphoxide and trichloroacetic acid were also assessed as buffer controls. Exposure of phosphatidylserine was then evaluated by staining with annexinV.

### Statistical analysis

Experiments were performed a minimum of three independent times. Data were analyzed with GraphPad Prism, version 4 (GraphPad, San Diego, CA). Comparisons between treatments were performed by one-way ANOVA. Statistical significance was tested at p<0.05 as the critical value. Data are presented as the mean ± SEM.

## Supporting Information

Figures S1 **Contig alignments between sequenced RNA fragments and 16S rRNA, 23S rRNA or tRNA^Asp^.** Contigs were generated using Vector NTI software to align sequences from the cloned RNA fragments with the respective H37Rv sequences obtained from Tuberculist. Depicted below are the contigs obtained for 16S rRNA (A), 23S rRNA (B) or tRNA^Asp^ (C).
(PPTX)

Figure S2 **Extracellular mycobacterial RNA fragments accumulate in the CF with similar kinetics as the rest of the mycobacterial secretome.** Low passage *M. tuberculosis* H37Rv was thoroughly washed three times in GAS media by centrifuging at 3000 rpm and decanting the supernatant. At different time points (indicated below figure) CF was harvested, filtered through a 0.2 µm filter and the CF was concentrated by centrifuging at 3000 rpm in an Amicon with 10 kDa cutoff membrane. Protein concentration was determined by BCA assay and 4.8 µg/lane were analyzed by SDS-PAGE plus silver (left gel) or ethidium bromide (right gel) staining.
(PPTX)
