# Supplementary figures and images for: Stable Extracellular RNA Fragments of Mycobacterium tuberculosis Induce Early Apoptosis in Human Monocytes via a Caspase-8 Dependent Mechanism
Source: PLoS One. 2012 Jan 9;7(1):e29970. doi: 10.1371/journal.pone.0029970 (PMC3253812; doi:10.1371/journal.pone.0029970)

## Slide 1
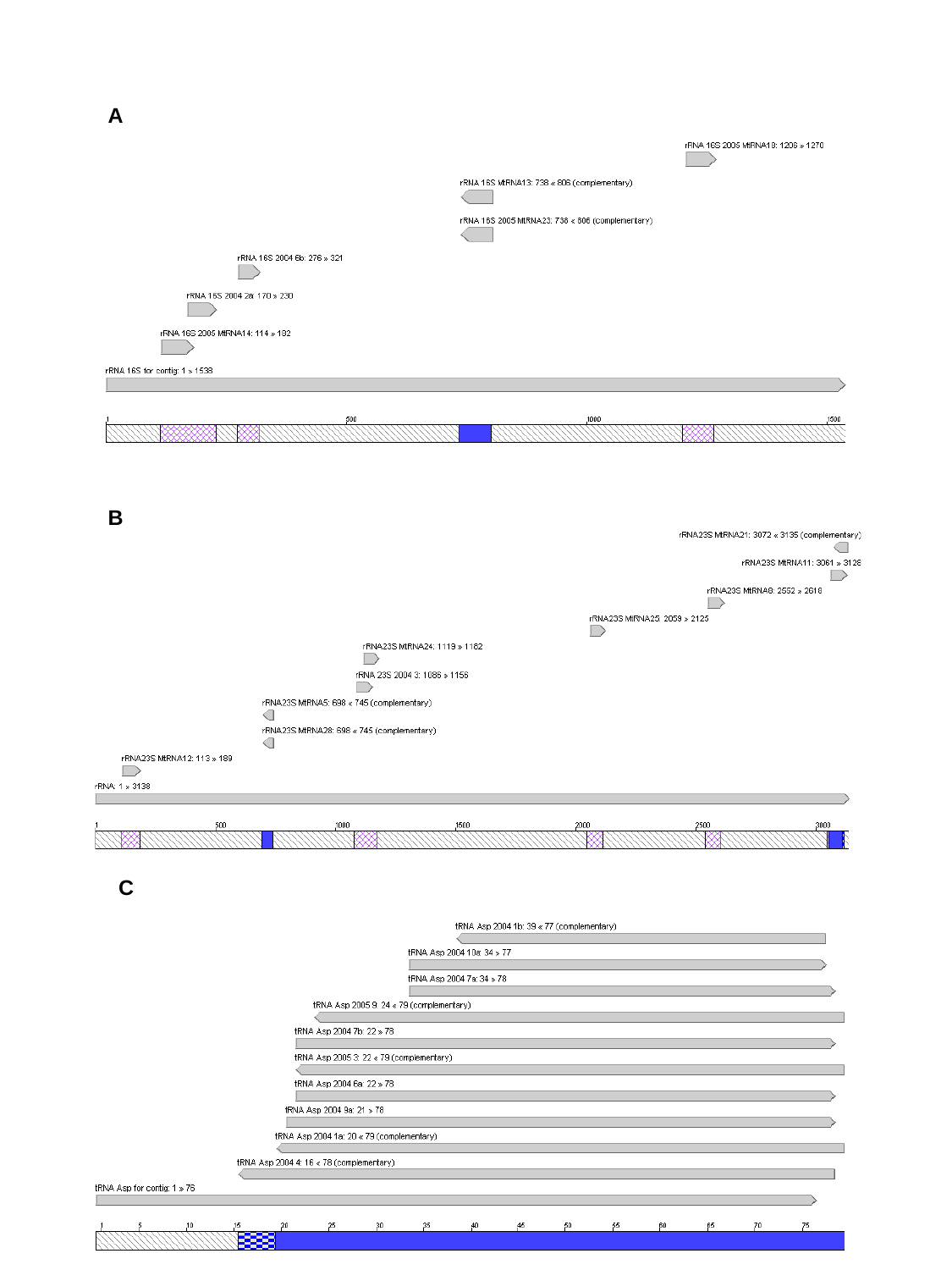

A
B
C

Supplement: Figures S1 — Contig alignments between sequenced RNA fragments and 16S rRNA, 23S rRNA or tRNAAsp. Contigs were generated using Vector NTI software to align sequences from the cloned RNA fragments with the respective H37Rv sequences obtained from Tuberculist. Depicted below are the contigs obtained for 16S rRNA (A), 23S rRNA (B) or tRNAAsp (C). (PPTX) [file pone.0029970.s001.pptx]

## Slide 1
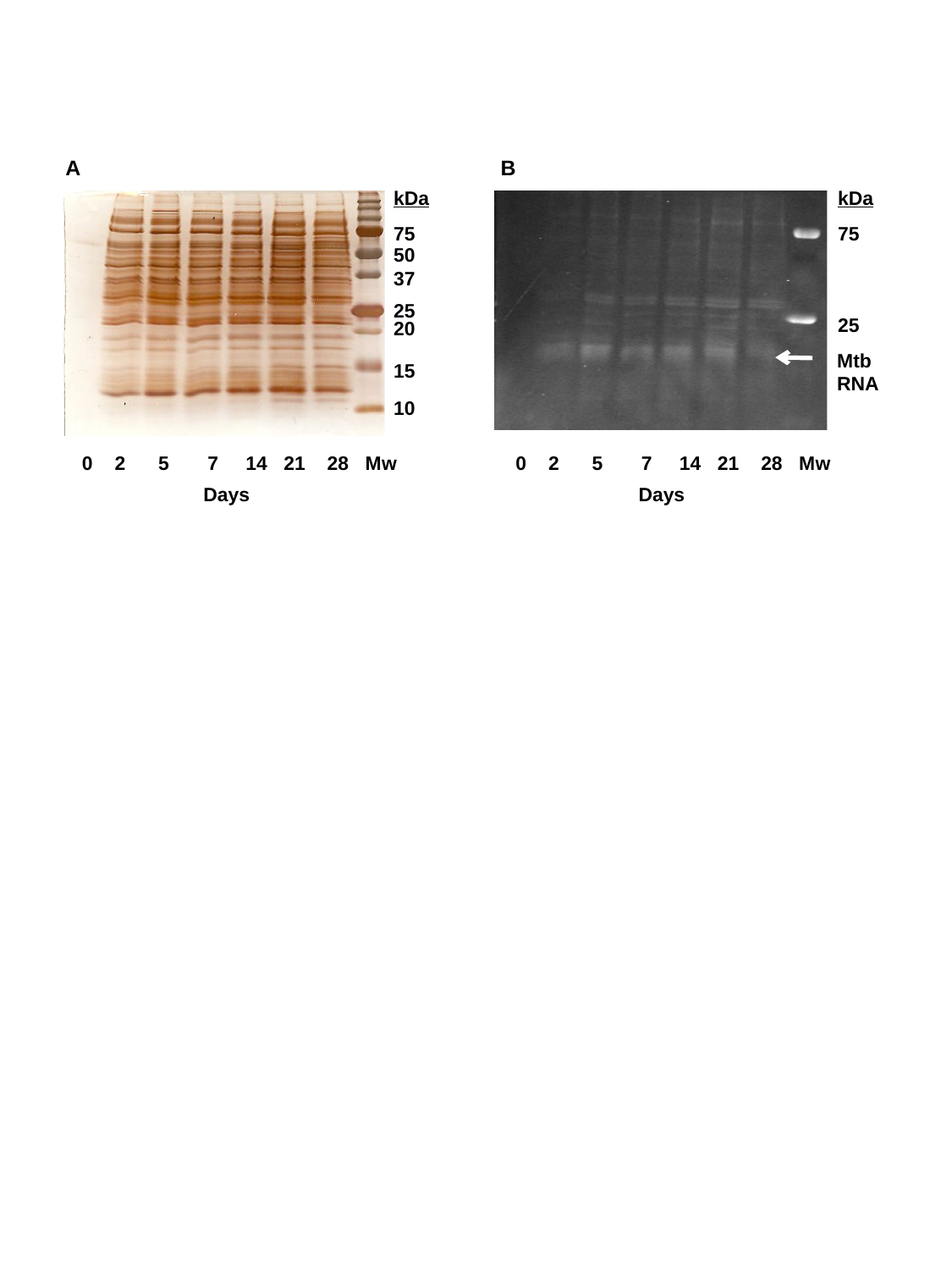

A
B
kDa
kDa
75
75
50
37
25
25
20
Mtb
RNA
15
10
0 2 5 7 14 21 28 Mw
0 2 5 7 14 21 28 Mw
Days
Days

Supplement: Figure S2 — Extracellular mycobacterial RNA fragments accumulate in the CF with similar kinetics as the rest of the mycobacterial secretome. Low passage M. tuberculosis H37Rv was thoroughly washed three times in GAS media by centrifuging at 3000 rpm and decanting the supernatant. At different time points (indicated below figure) CF was harvested, filtered through a 0.2 µm filter and the CF was concentrated by centrifuging at 3000 rpm in an Amicon with 10 kDa cutoff membrane. Protein concentration was determined by BCA assay and 4.8 µg/lane were analyzed by SDS-PAGE plus silver (left gel) or ethidium bromide (right gel) staining. (PPTX) [file pone.0029970.s002.pptx]
